# Supplementary figures and images for: Burst firing is required for induction of Hebbian LTP at lateral perforant path to hippocampal granule cell synapses
Source: Mol Brain. 2023 May 22;16:45. doi: 10.1186/s13041-023-01034-w (PMC10204231; doi:10.1186/s13041-023-01034-w)

Fig. S1

A

RS

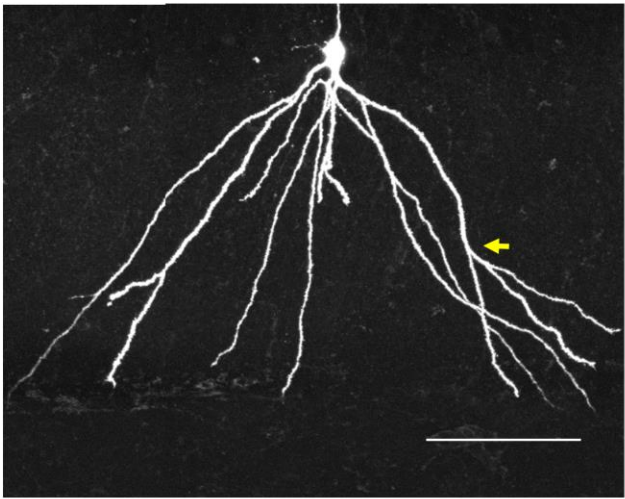

BS

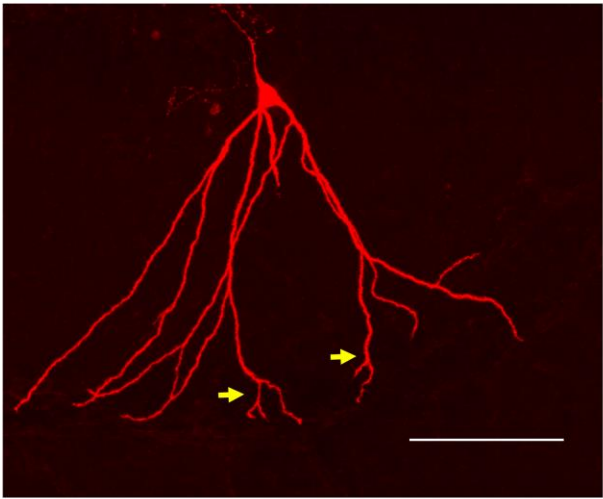

Fig. S2

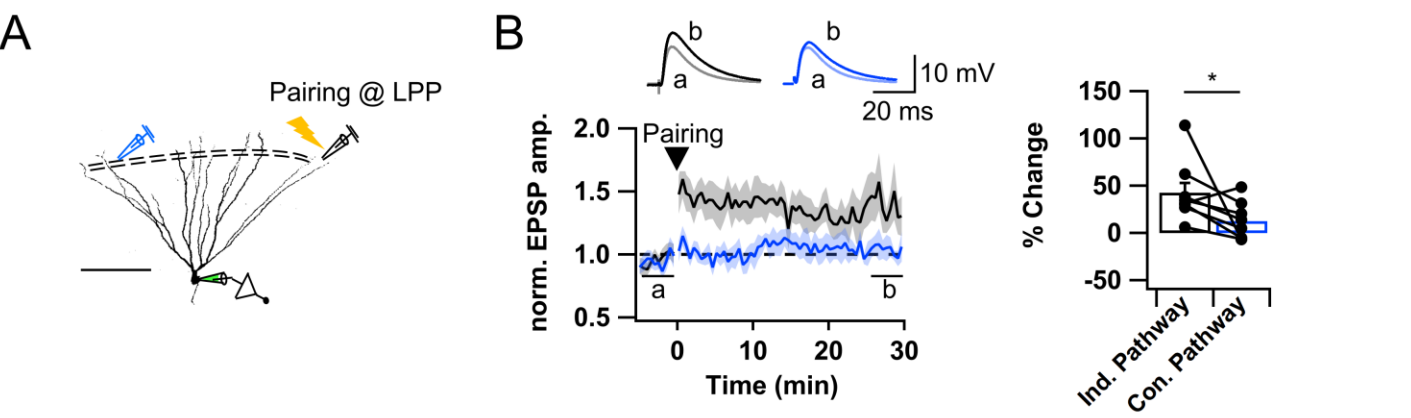

Fig. S3

A

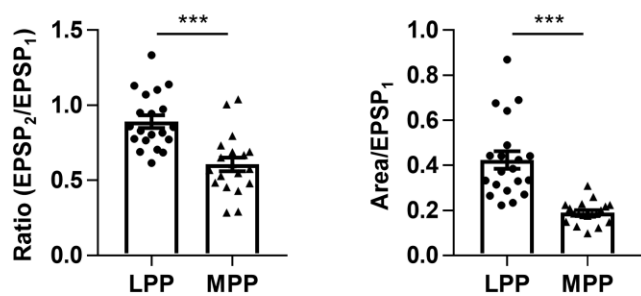

B

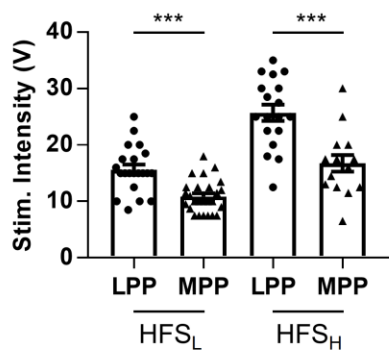

C

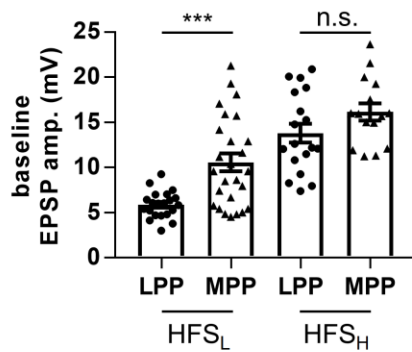

D

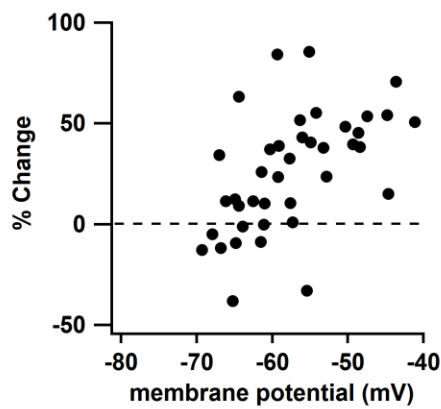

**Fig. S4**

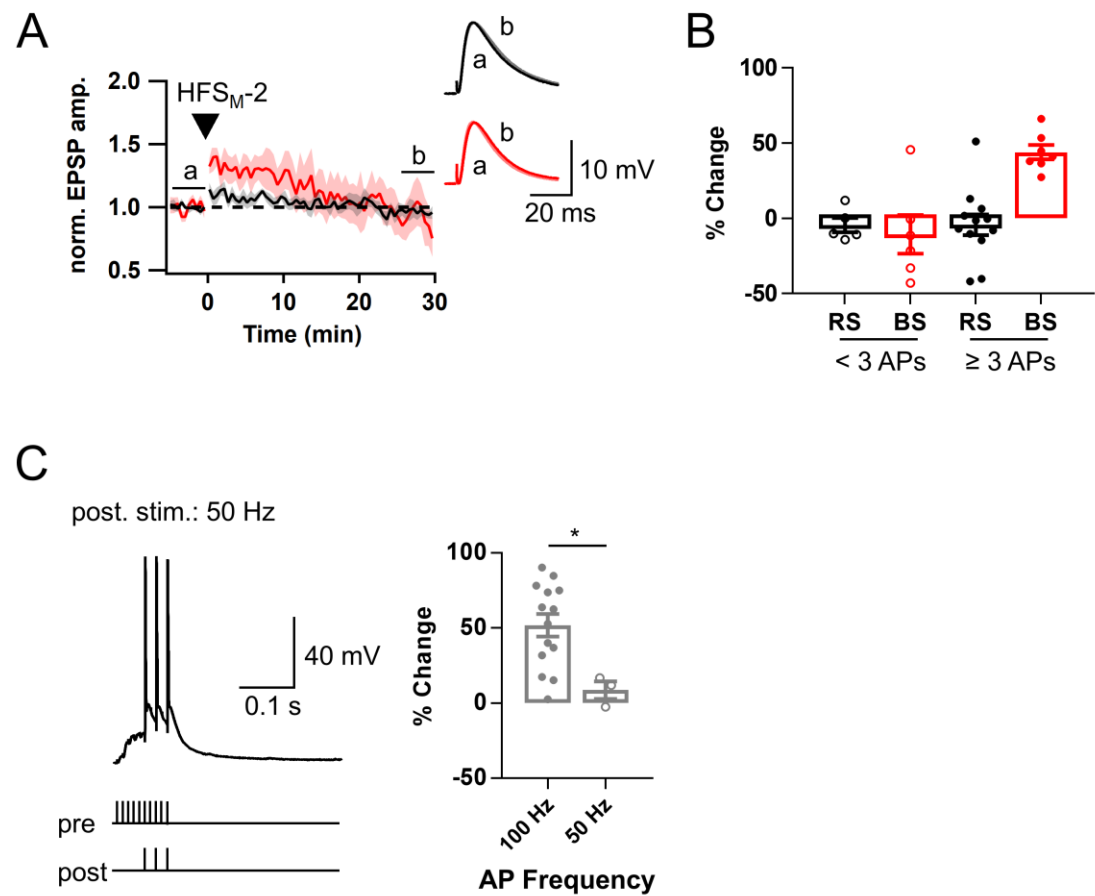

**Fig. S5**

**A**

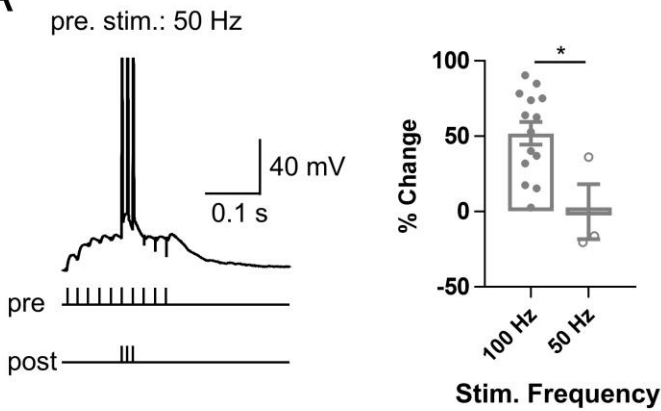

**B**

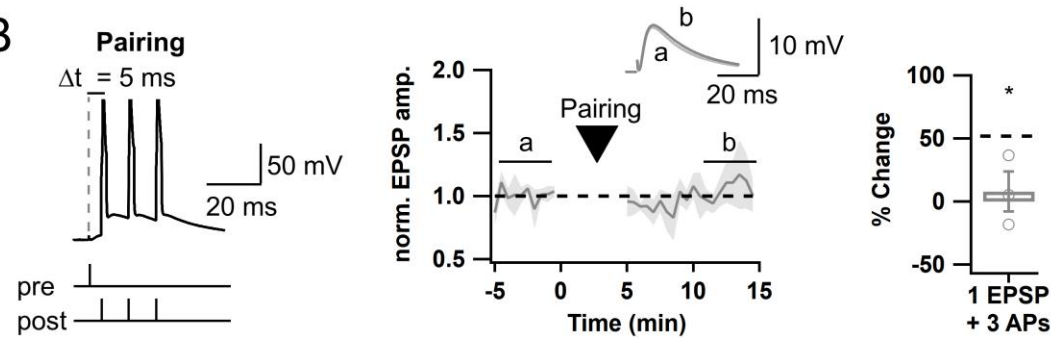

Supplement: Supplementary file 1 — Additional file 1: Fig. S1. Representative biocytin-filled RS-and BS-GC. Yellow arrows indicate the maximal dendritic branching points. Scale bar, 100 μm. Fig. S2. Input specificity of postsynaptic AP-dependent LTPat LPP synapses. A: Two stimulation electrodes were placed at outer molecular layer. B: Baseline EPSPs were monitored at the two electrodes. HFSL was delivered to one of two electrodesbut not to the other electrode. Pairing protocolwas applied at the induction pathway. Note that LTPAP was induced only at the synapse that underwent HFSL. Shades and error bars, S.E.M. *p < 0.05. Fig. S3. Stimulation Intensities and baseline EPSP amplitudes to evoke sub- or suprathreshold voltage responses at MPP and LPP synapses. A: Compared to MPP-EPSPs, LPP-EPSPs were significantly higher in paired pulse ratioand in the normalized area of subthreshold EPSP summationto EPSP1 amplitude. B: Mean stimulation intensities used for HFSL and HFSH at MPP and LPP synapses. Both mean intensities for HFSLand HFSHwere significantly stronger at LPP-GCs than MPP-GCs. C: Baseline amplitudes of EPSP evoked by HFSL and HFSH at MPP and LPP synapses. Significantly larger baseline EPSP amplitudes were required at MPP-GCs than LPP-GCs in order to elicit subthreshold responses. But, it was not significant to elicit 3 APs responses. D: Plot of LTPsub magnitude as a function of peak membrane potential of EPSP summation. Error bars, S.E.M. ***p < 0.001. n.s., not significant.. Fig. S4. Conditions for LTPAP induction. A: Time course of normalized EPSP before and after HFSM-2. HFSM-2 is defined by HFS eliciting 1 or 2 APs. Note that LTP was not maintained not only in RSbut also in BS. B: Magnitudes of LTPAP induced by HFSM or HFSH in RS and GS. C: Dependence of LTPAP on the postsynaptic AP frequency. When the post-synaptic AP bursts were elicited at 50 Hz instead of 100 Hz in the pairing protocol, LTPAP was not induced. Shades and error bars, S.E.M. *p < 0.05. Fig. S5. A: Dependence of LTPAP on the s [file 13041_2023_1034_MOESM1_ESM.pdf]
